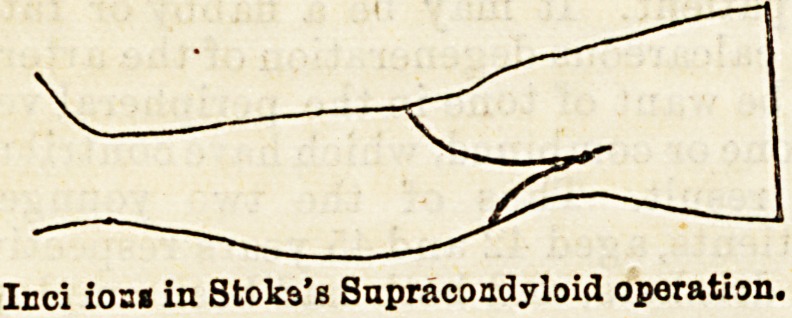# Senile Gangrene

**Published:** 1893-05-06

**Authors:** 


### HUDDERSFIELD INFIRMARY.

### SENILE GANGRENE.

During the year 1892 eighteen cases of gangrene were treated in the wards of the Huddersfield Infirmary, and in each case the gangrene was the ailment for which the patient sought admission. Three of the patients were diabetic, the ages of these being 55, 57, and 62 years respectively, and of them one died nine days after admission, one six days after admission, whilst the youngest declined operation, and went home after thirteen days' treatment. Of the two cases that died, in one amputation of the leg was done for gangrene of the toes.

The average age of the remaining fifteen cases, of whom only two were women, was 54·8 years, ranging from 42 years in the youngest to 68 in the oldest. Five of the cases were not 50 years old, and five not sixty; and of the five 60 years or more old, only two were more than 61. Hence, it must be confessed that the orthodox term senile gangrene is somewhat of a misnomer, for it has to include cases to which the definition senile, as indicating old age, does not apply. With this qualification the definition has its uses, for it reminds us that gangrene of the type we are considering is not strictly a local disease, that, in fact, the death of a toe or toes is an expression of a degraded constitutional state, which has to be taken into consideration in all attempts of the surgeon to relieve his patient. It may be a flabby or fatty heart; it may be calcareous degeneration of the arterial walls; or it may be want of tone in the peripheral vessels and tissues, alone or combined, which have contributed to the untoward result. Thus of the two youngest of the fifteen patients, aged 42 and 45 years respectively, both had been drinkers, and both had been employed where drink was easy to obtain, one at a public-house, the other at a brewery. These preliminary considerations cannot be overlooked in any attempt to discuss the treatment of gangrene, for it is to the condition of the patient that the surgeon must look, not only to decide the treatment to be adopted, but for any solace he wishes to have if his cases do not end so well as he would desire.

Of the fifteen cases five died, seven recovered, and three are still under treatment. In three of the fatal cases amputation was performed in the lower third of the thigh, death in one case being due to hypostatic pneumonia, in another to syncope forty-eight hours after operation, when the patient was apparently doing well. Of the seven cases that recovered in two only was operation done, in one case in the lower third of the thigh, in the other in the lower third of the leg.

Mr. Knaggs considers that when the gangrene is confined to the toes and shows no disposition to spread no operation should be thought of, but should the gangrene spread into the foot and be inclined to extend then amputation should be performed, though, adds Mr. Knaggs, I should be very chary indeed in operating whenever it was associated either with renal disease or diabetes. Dr. Clarke advocates amputation whenever and as soon as the gangrene spreads beyond the toe or toes originally attacked, and he thinks many lives might be saved by the adoption of this rule.

Dr. Martin is generally in favour of amputation, and would operate at once if the condition of the patient is satisfactory. Mr. Porritt, Dr. Irving, Dr. Robinson, and Dr. Wright adopt the expectant method more closely. Dr. Irving for some time has left the cases severely alone, so far as operative interference is concerned, as any attempt to cut away a portion of a toe or a whole toe, even when it appears ready to come away easily, sets up a good deal of pain, and disturbs the parts. Dr. Irving has never amputated a leg for senile gangrene of the foot, and so far has had no death among his infirmary cases. Dr. Robinson considers the condition of the patient more of a guide for operation than the extent of the gangrene, and if the patient is in a good condition for his age, if the signs of calcareous degeneration are limited, he would give nature a larger margin of time to effect separation of the mortified part. Where the gangrene is the result of a life of excess Dr. Robinson would delay operation as long as possible, though if the gangrene begins to spread actively amputation probably gives the best chance.

Dr. Wright considers the less we interfere, the better for the patient; and he is very chary of operating unless it seems to be called for by septic absorption.

Operation being demanded, at what point should the limb be removed? The opinion of the staff of the Huddersfield Infirmary is unanimously in favour of getting a long distance from the gangrenous tissues, and though Dr. Clarke would not amputate at the lower third of the thigh indiscriminately the other members of the staff would do so, preferring, in cases where amputation is decided upon, to take the risk of the extra shock among healthier tissues nearer the body, for the sake of the less likelihood of sloughing of the stump, which may occur when the amputation is performed nearer the gangrenous parts. Dr. Wright favours Stokes's supracondyloid operation, whilst Dr. Robinson thinks the circular operation is well adapted for these cases.

Inci ions in Stoke's Supracondyloid operation.

Having decided upon the line of treatment, operative or expectant, we will assume that the latter has been adopted. Four indications are, as far as possible to be carried out: (1) To keep up the strength and improve the general condition of the patient; (2) to relieve pain and procure sleep; (3) to favour the separation of dead tissue; (4) to prevent septic absorption and local inflammatory action around the gangrenous tissues. The diet must be suited to the digestive powers of the patient, but should be at all times as nourishing as possible. If solid food can be taken it should not be withheld, and the addition of beef tea, eggs, and plenty of milk will be of use. It must be remembered that separation of dead tissues by natural powers, perhaps weakened and enervated, is slow and tedious, making, with the attendant pain, a continuous demand on the patient's capabilities. In not a few cases stimulants are called for, and in some must be given with no unsparing hand, although if the process is proceeding without local inflammatory action there can be no doubt that nourishing food is of more value than stimulating alcohol.

Moreover, the patient should be in bed in a well-ventilated room. The stench sometimes, in spite of dressings, is not to be kept down; whilst, on the other hand, abundant pure air has a good influence on the nutrition of the patient. Tonics such as the vegetable bitters, to improve the appetite, with or without iron, quinine, nux vomica, and the mineral acids, are also of use by promoting a better state of the general health.

Of all drugs, however, opium is of the most general use, in doses of from half a grain upwards. It should be given in sufficient doses to procure sleep and relieve pain. It is best given in pill at bed-time, though, if pain is severe, it is not to be withheld during the day. The hypodermic injection of morphia is not used so extensively as the opium pill, which in many cases has been the only medicine given. In a case where even half a grain of morphia failed to relieve, Dr. Wright found hyoscine a most efficient substitute. Mr. Knaggs sometimes prescribes opium in the form of Dover's powder. If opium causes constipation, some aperient may be needed to relieve the constipation.

In the early stages of the affection the foot is elevated by placing the leg on a pillow. The parts are dredged with iodoform, and some dry dressing, *e.g.*, iodoform gauze, perchloride wool, or simple wadding, is applied once a day. Dr. Irving paints the affected parts with compound tincture of benzoin, and then covers the foot with cotton wool or some antiseptic wool. Any interference with the gangrenous toe or toes is most carefully avoided, as likely to cause the gangrene to spread, the most perfect rest and quiet being maintained, whilst, at the same time, the foot is covered with an abundance of cotton wool, in order that all the heat that is possible may be retained in the parts.

When, as indicated by the appearance of the line of demarcation, separation of the parts is commencing, the dry dressings are usually changed to hot boracic fomentations, made by soaking boracic lint in boiling water. These are changed by the nurse every two hours, india-rubber tissue and cotton wadding being bandaged over the hot boracic lint. Boracic fomentations are preferred to charcoal poultices. They are cleaner, lighter for the patient to bear, are easily applied by the nurse, and as an antiseptic application are superior to the poultices.

It is during the process of separation that local suppuration, thecal abscess, with perhaps sloughing of the plantar fascia, are to be feared. The bones throw off their dead portions without trouble, but the tendons sometimes retract in their sheaths before they have quite cast off the dead remnant, and the portion of slough carried out of sight into the sheath may set up secondary abscess. If this occur the pus, if not in large quantity, may be squeezed gently out through the open tendon sheath night and morning; but if at all abundant it is better to incise the abscess, give free exit to the pus, and treat it as an abscess on the usual surgical principles.

When the gangrenous part has separated an open sore may remain. Frequently the healing of the living tissue is contemporaneous with the separation of the dead; but if a granulating surface be left, it must be treated on the usual principles.

## Figures and Tables

**Figure f1:**